# Impact of online learning on sense of belonging among first year clinical health students during COVID-19: student and academic perspectives

**DOI:** 10.1186/s12909-023-04061-2

**Published:** 2023-02-08

**Authors:** Clarice Tang, Liz Thyer, Rosalind Bye, Belinda Kenny, Nikki Tulliani, Nicole Peel, Rebecca Gordon, Stefania Penkala, Caterina Tannous, Yu-Ting Sun, Leigha Dark

**Affiliations:** School of Health Sciences, Western Sydney University, Sydney, NSW Australia

**Keywords:** Health, Higher education, Mixed methods, Sense of belonging, Situated learning theory, Undergraduate

## Abstract

**Background:**

The need to belong is a fundamental human desire that provides the basis for relationships and community; it provides a sense of security that enables growth and development. This sense of belonging is pivotal to new University students, indeed, without it, students are at greater risk of failing or withdrawing from their studies. Yet developing a sense of belonging within a new cohort is complex and multi-faceted and further complicated by a sudden shift away from in-person to online learning. Using the situated-learning framework, our study explores first year clinical health students’ sense of belonging in the context of the rapid transition to online learning because of the COVID-19 pandemic.

**Methods:**

We utilised a current mixed-method approach including a survey incorporating previously validated tools, demographic and open-ended qualitative questions. Data was also gathered from three focus groups: two dedicated student groups and one academic focus group. Qualitative data was subjected to thematic analysis whilst descriptive statistics were used to describe the quantitative data.

**Results:**

179 first year students complete the survey and four students, and five academics were involved in the focus groups. All participants were from clinical health science courses at an Australian university. Our qualitative results indicated a global theme of: Navigating belonging during the COVID-19 crisis: a shared responsibility; with four organising themes describing (1) dimensions of belonging, (2) individual experiences and challenges, (3) reconceptualising teaching and learning, and (4) relationships are central to belonging.

**Conclusion:**

While the rapid transition to online learning did not greatly impact knowledge acquisition of first-year students in this cohort, the lack of sense of belonging highlights the need for further research into development of this essential aspect of learning in the online domain. Although contextualised in the COVID-19 pandemic, it became clear that the findings will remain relevant beyond the current situation, as a student’s need to belong will always be present in the face of challenges or change.

**Supplementary Information:**

The online version contains supplementary material available at 10.1186/s12909-023-04061-2.

## Background

The importance of belonging is gaining wider recognition within higher education [1]. Defined as interpersonal relatedness [2] and "a subjective feeling of value and respect derived from a reciprocal relationship” [3], promoting a sense of belonging is a key enabler to improve transition [4], retention and completion for students in higher education [5]. Sense of belonging is typically developed through regular in-person interactions between students and staff [2, 6, 7] fostering shared values and ensuring students feel validated. This is particularly pertinent to transition, for example commencing university, where shared experiences can encourage reciprocal student-peer interactions and social connection that are protective and motivating when students face later challenges [8, 9]. Consequently, a focus on developing sense of belonging is crucial during the first year at university [2, 7]. A “whole-of-institution and whole-of-student” approach that offers activities to promote transition and connection to university is considered best practice with importance placed on non-traditional groups (first-in-family, low socioeconomic background, part-time enrolment) [5].

When the COVID-19 pandemic began in 2020, universities faced major challenges developing effective ways to promote students’ sense of belonging online. For many students, this unplanned move to an online environment was threatening and compounded their lack of confidence and loneliness [6]. Even prior to COVID-19, literature suggested that students struggled to connect through online learning communities impacting engagement and leading to feelings of isolation [10]; historically, attrition rates for online learners are higher compared to those who undertake their studies in-person [10].

Recent studies have shown that commitment from academics and good design of online curriculum, learning activities and assessments, combined with technical support can assist retention and development of sense of belonging [11–13]. However, while online technology enabled students to be present throughout the pandemic, and engage with learning content, student mental health, well-being and sense of belonging were bigger issues that may not have been met. A study of online learning by Parker et al. [14], found intra- and inter-personal communication, adaptability, and stress management skills were important when developing content so students felt connected, respected, and accepted. While a qualitative study conducted by Thomas and colleagues [13] explored how online platforms could foster a sense of belonging. Unfortunately, neither defined how the online space could facilitate sense of belonging. While there has been some literature evaluating impact of online learning on students' sense of belonging [6, 9], none of these studies investigated clinical health science students. Promoting a sense of belonging among first year clinical health science students is especially important for developing positive motivational attitudes, professional identity [15, 16], and professional peer relationships with future colleagues [17]. There are limited studies that have examined impact of online platforms on the development of sense of belonging amongst undergraduate first year clinical health science students and, as yet, none have provided conclusive guidance.

The aim of this study was to explore clinical health students’ sense of belonging during their first year of university and determine whether sense of belonging varied across different student populations. This study further explored academics’ perspectives on online learning strategies to facilitate student sense of belonging.

## Methods

This study utilised a mixed-method approach [18, 19] in which first year students were asked to participate in a survey and subsequently a focus group. Focus groups with academics were also conducted. Academics in this study refer to staff involved in design and/or delivery of relevant first-year subjects. Themes from the focus groups were triangulated [20] with the findings from the survey to provide a comprehensive picture about how online learning impacted overall sense of belonging among first year clinical health science students. This study obtained ethics approval from the Western Sydney University Ethics Committee (H13857).

### Theoretical framework

Situated Learning Theory (SLT) was used as a framework to consider the results of our study. SLT has been described as learning that is epitomised by the social context including spaces outside the classroom; co-construction culture between those with more and those with less developed knowledge; and the often-unintentional learning that occurs through active learner participation [21]. SLT has been widely used within health professional education [22], either as a component of a broader framework [23], to investigate interprofessional learning [24], consider specific aspects of teaching and learning such as assessment [25], and the role of sense of belonging within the learning context [26]. Moving beyond the best-known aspect of the theory, Communities of Practice (CoP), we considered how aspects of Legitimate Peripheral Participation (LPP) and identity could be used to conceptualise our findings and took account of Wegner’s call to reflect upon purpose, stance, technical terms, and boundaries of the theory [27].

### Online student cross-sectional survey

All students enrolled in first year health profession-specific subjects across seven clinical health science Bachelor-degree courses, at the host university in Sydney, Australia, were eligible to participate in the online survey which was disseminated electronically from May 2020 to October 2020. First year subjects were specifically chosen for eligibility into the study as they are purposefully designed with introductory content for each profession. These introductory subjects have also only been taught previously via in-person learning. In 2020, students completed three weeks of in-person learning before rapidly transitioning to an online learning model for the remainder of the semester due to COVID-19. Students were invited to participate in the survey via email.

The online survey was a 25-item questionnaire, which included eight demographic questions, seven questions about their overall sense of belonging to the University [1], eight questions about overall impact of online learning on their perception of the course and two further open-ended questions about facilitators for and barriers to fostering sense of belonging via an online learning method. Sense of belonging was evaluated using the 7-item Sense of Belonging Scale developed by Imperial College London [1], which has been adapted based on the previously validated Sense of Belonging in Higher Education Scale [2] and the Harvard Panorama Student Perception Survey Scale [28]. All items were rated on a 5-point Likert scale (1: not at all, 2: slightly, 3: somewhat, 4: quite and 5: extreme), with higher scores indicating a greater sense of belonging [1]. Using the same 5-point Likert scale, participants rated impact of online learning on their overall perception of the course. General demographic information included i) age, ii) sex, iii) educational status prior to current enrolment and iv) if they were connected to other students within the course, either through previous acquaintances or social media. Survey drafts underwent repeated review by members of the research team to ensure readability, face validity and structure and followed the guidance in relation to amendments to the survey [1]. Following refinement, the survey was circulated to students via the online survey platform Qualtrics (Qualtrics, Provo, UT, 2020).

Data were entered into IBM SPSS version 26 (Chicago, USA). Nominal and ordinal data were coded for statistical analysis. Categorical data were reported as sample size and percentages and non-parametric data without normal distribution (i.e., age) were reported as median and interquartile range (IQR). To determine if prior experience in higher education influenced sense of belonging to the university, connectiveness to their chosen course, and/or intention to continue university studies, a Mann–Whitney U rank test between groups (students with prior university degree experience versus students with only high school education experience) was conducted.

Open survey responses were analysed using a descriptive content approach [29]. Data were coded into meaning units and consolidated into unique categories through a systematic analysis. Patterns and themes were identified through systematic analysis of the open-ended responses. The process was undertaken by two researchers (LT and LD) independently with any discrepancies being resolved through discussion and inclusion of a third reviewer (CT)[30].

### Student and academic focus groups

Focus groups were conducted with both academics and students to obtain rich and meaningful data about the impact of online learning/teaching on overall perceived sense of belonging among students [31]. Purposive sampling was used to allow any student from the first-year cohort, and any staff members teaching that cohort, to voluntarily participate in the focus groups. This method of sampling is widely adopted in qualitative and mixed methods research to gain participants who are experienced with, or knowledgeable about, the research area, and willing to share their perspectives [32]. In this case, all staff and students involved in the first-year profession-specific subjects were invited to take part, with the aim of recruiting five to sixparticipants per group. This size corresponds with recommendations for smaller focus groups investigating topics well known to participants and the studies investigating their experiences or behaviour [31]. The size allows increased participant engagement and ease of moderation by focus group facilitators [31].

For students, following completion of the online survey, they were asked to indicate interest to participate in a voluntary online focus group via Zoom (Zoom Video Communications Inc, 2016). Following an expression of interest, written informed consent was electronically gained and recorded prior to the online focus group running. For academic focus groups, all academics, including casual (those not employed in an on-going capacity) team members, who taught into the relevant subjects and delivered a minimum of 20% of the online content, were invited to participate via email. Interested academics gave electronic written informed consent prior to participating in online focus groups via Zoom. No compensation was provided to participants.

All focus groups were facilitated by independent, experienced moderators with no links to participants to minimise likelihood of bias. The online format was necessary due to local government public health orders and although a feature of social research approaches for some time [33] becoming more common since the pandemic [34]. Topics discussed during focus groups were guided by a semi-structured interview guide [31] including questions related to impact and effectiveness of the online mode on student engagement, strategies implemented to create a sense of student belonging and their effectiveness, and the challenges to belonging posed by the online platform.

The focus groups were audio-taped and transcribed verbatim. Field notes were also taken before, during and after the focus group to capture expression of body language from individual participants and any additional points that were provided before or after the focus group. Final transcripts were thematically analysed by four members of the research team (BK, NT, RB and NP) using thematic analysis methods outlined by Attride-Stirling [35]. Each interview transcript was first read in its entirety to gain an overall sense of the participants’ perceptions. Each data set was initially read, and then the process of assigning open codes through identification and isolation of words or statements that reflect meaning were independently completed for all data. Coders for each data set compared coding and any differences were resolved by returning to the transcript and by consensus. Following independent coding, the two researchers analysing the student data, and the two analysing the academic data, collaborated to identify themes for each aspect of the study. The research team then met to discuss these findings, and commonality was found across the student and academic data sets. Hence, the research team decided that the data would be combined to present themes inclusive of both student and academic perspectives. Themes were documented with the perspectives of students and academics presented. Other members of the research team also contributed and agreed to the final identification of themes of the study, and the diagrammatic representation of the thematic network was developed, consistent with methods outlined by Attride-Stirling [35] whereby a global theme, organising themes and basic themes are developed and related pictorially. The results from the descriptive content analysis were also integrated with thematic networking [35] to present themes that were inclusive of data from both surveys and focus groups.

## Results

### Online student cross-sectional survey

#### Demographic characteristics

A total of 179 out of the possible 663 students (27% completion) completed the online survey in June 2020. Median age of students was 19 years (IQR 18–28 years) and there were approximately three times as many females as males (Table 1), reflective of the undergraduate health sciences cohort (70% female). Student numbers were also reflective of the broader enrolment numbers in the programs (i.e., occupational therapy is the largest program). Just over half (53%; *n* = 94) of students had no prior experience in undertaking a Bachelor degree, and 76% of students had not completed any online courses prior to enrolment.

**Table 1:** Demographic characteristics

Demographic characteristics	*N* = 179
Median age, years (IQR)	19 (18 – 28)
Sex, n (%)	
Female	139 (78)
Male	29 (16)
Unknown	11 (6)
Program of study, n (%)	
Occupational Therapy	47 (26)
Paramedicine	38 (21)
Physiotherapy	30 (17)
Speech Pathology	24 (13)
Recreational Therapy	14 (8)
Podiatry	12 (7)
Undisclosed	9 (5)
Traditional Chinese Medicine	5 (3)
Highest prior educational experience, n (%)	
Completed high school, college or vocational institute	94 (53)
Started but did not complete Bachelor degree	40 (22)
Completed Bachelor degree or higher	27 (15)
Others	18 (10)
Connected with other students within program via social media, n (%)	
Yes	126 (70)
No	46 (26)
Undisclosed	7 (4)
Prior experience in completing online courses, n (%)	
No	136 (76)
Yes	36 (20)
Undisclosed	7 (4)

### Quantitative results to the sense of belonging questionnaire

In terms of students’ sense of belonging to the university, the majority felt ‘quite’ or ‘extremely’ happy with their choice of university (74%) and felt ‘quite’ or ‘extremely’ welcomed by the university (68%). While most students felt respected by both staff (70%) and students (60%) at the university, students reported less connectiveness (23.5%) to the university. Only 20% of students reported they felt they were understood as an individual, and only 13% felt they ‘quite’ or ‘extremely’ mattered to others at the university (Table 2).

**Table 2:** Online learning and Sense of Belonging to the University [1]

Question	Responses on 5-point Likert Scale, n (%)						Mean ranks		Between group differences *U*, *p*-value
	No response	1: Not at all	2: Slightly	3: Somewhat	4: Quite	5: Extremely	Prior Exp *N* = 65	No prior Exp *N* = 91	
How well do people understand you as a person?	12 (6.7)	20 (11.2)	53 (29.6)	58 (32.4)	24 (13.4)	12 (6.7)	79.2	78.0	2914.5, 0.9
How connected do you feel to the University?	12 (6.7)	21(11.7)	53 (29.6)	51 (28.5)	37 (20.7)	5 (2.8)	84.5	74.2	2567.5, 0.1
How welcoming have you found University to be?	12 (6.7)	2 (1.1)	10 (5.6)	34 (19.0)	92 (51.4)	29 (16.2)	79.1	78.1	2919.0, 0.9
How much respect do other students show towards you?	13 (7.3)	0 (0)	9 (5.0)	49 (27.4)	82 (45.8)	26 (14.5)	68.7	85.5	^a^2318.0, 0.01
How much respect do staff at the University show towards you?	13 (7.3)	0 (0)	8 (4.5)	32 (17.9)	87 (48.6)	39 (21.8)	75.6	80.6	2770.5, 0.5
How much do you matter to others at the University?	13 (7.3)	30 (16.8)	45 (25.1)	67 (37.4)	19 (10.6)	5 (2.8)	74.3	80.6	2674.0, 0.4
How happy are you with your choice to be a student at the University?	12 (6.7)	2 (1.1)	11 (6.1)	21 (11.7)	92 (51.4)	41 (22.9)	83.7	74.8	2620.0, 0.2

*Exp* Experience in higher education

^a^represents significant differences

*p* < 0.05

Table 3 shows how the online learning experiences impacted on students’ perception of the course; 27% of students felt ‘quite’ or ‘extremely’ connected to staff while 16% of students felt ‘quite’ or ‘extremely’ connected to other students. While 49% of students rated 4 and above for the level of respect that they received from other students and their contribution towards the subject, students who had prior higher education felt *less* respected than students who had no prior higher education (*p* = 0.03). When asked how the online subject had contributed to understanding, knowledge/skills in their chosen health profession, about half of the students rated the online subject highly (rating 4 and above). Students who had prior higher education indicated higher ratings of understanding and knowledge/skills compared to students without prior higher education (*p* = 0.07 and *p* = 0.03 respectively). There was also a significantly higher proportion of students with no prior higher education who identified the online learning experience as either ‘quite’ or ‘extremely’ likely to impact their intention to continue with their current course (*p* = 0.001).

**Table 3:** Impact of online profession-specific subject on perception of the course

Question	Responses on 5-point Likert Scale, n (%)						Mean ranks		Between group differences *U*, *p*-value
	No response	1: Not at all	2: Slightly	3: Somewhat	4: Quite	5: Extremely	Prior Exp *N* = 61	No prior Exp *N* = 87	
How much has the online learning experience facilitated you to feel connected with staff in your course?	20 (11.2)	19(10.6)	39 (21.8)	52 (29.1)	39 (21.8)	10 (5.6)	80.5	70.2	2287.0, 0.1
How much has the online learning experience facilitated you to feel connected with other students in the course?	20 (11.2)	42 (23.5)	57 (31.8)	32 (17.9)	21 (11.7)	7 (3.9)	76.5	73.1	2530.5, 0.6
How much respect do other students in the subject show towards you?	20 (11.2)	0 (0)	20 (11.2)	52 (29.1)	65 (36.3)	22 (12.3)	65.8	80.6	*2124.0, 0.03
How well do you perceived your needs and contributions are respected in the subject?	20 (11.2)	4 (2.2)	24 (13.4)	43 (24.0)	68 (38.0)	20 (11.2)	74.3	74.6	2644.5, 0.97
How much has the subject developed your understanding of your chosen profession?	20 (11.2)	3 (1.7)	28 (15.6)	31 (17.3)	50 (27.9)	47 (26.3)	81.8	69.4	*2208.0, 0.07
How much has the subject developed your knowledge and skills in your chosen profession?	20 (11.2)	2 (1.1)	26 (14.5)	43 (24.0)	44 (24.6)	44 (24.6)	83.4	68.3	*2109.5, 0.03
How happy are you with your choice to be a student in your enrolled course?	21 (11.7)	4 (2.2)	13 (7.3)	20 (11.2)	66 (36.9)	55 (30.7)	83.1	67.7	*2061.5, 0.02
How have your online learning experiences impacted your intention to continue with your current course?	20 (11.2)	45 (25.1)	28 (15.6)	31 (17.3)	33 (18.4)	22 (12.3)	61.1	83.9	*1835.0, 0.001

### Qualitative results

Qualitative findings provided insight into experiences of staff and students during the rapid, unplanned transition to online learning. Student questionnaire responses included two open-ended questions expanding on enablers and barriers to sense of belonging. These yielded 145 enablers and 254 barriers to students’ feeling a sense of belonging. Data were subjected to qualitative content analysis by two authors and categories are presented in Additional file 1.

Three focus groups were conducted: two student sessions, each with two students enrolled in Speech Pathology and Paramedicine, and one academic session with five participants. Four full time academics and one casual academic participated from a total population of nine eligible academics. Using the processes described in the methods, focus group analysis was compared with the survey content analysis and the authors identified synergies between them. Findings were then integrated under a global theme, underpinned by organising and basic themes. The following themes reflect triangulation between academic and student focus group data in addition to survey responses.

#### Global theme—navigating belonging during the COVID-19 crisis: a shared responsibility

“We are in this together…making the best of this”

This theme explores sense of belonging creation during this period as a shared process, where participants perceived they worked together to get through the crisis. Students and academics encountered many challenges as they transitioned to online learning but despite hard times, were able to engage positively. The global theme revealed students and academics were *navigating belonging during the COVID-19 crisis*, and this journey was *a shared responsibility*. Both groups were working to achieve positive student engagement that would in turn create a sense of belonging in first-year students. A strong commitment of working hard to make the best out of this was mutually acknowledged**.**

Students perceived academics had done “a really good job at making sure we belonged…in those first few weeks that we were on campus but even more so probably while we were in Zoom” (Student-Astrid-Focus Group). Academics perceived students were actively engaged in making online learning work and were collegial and collaborative.

**Fig. 1:**
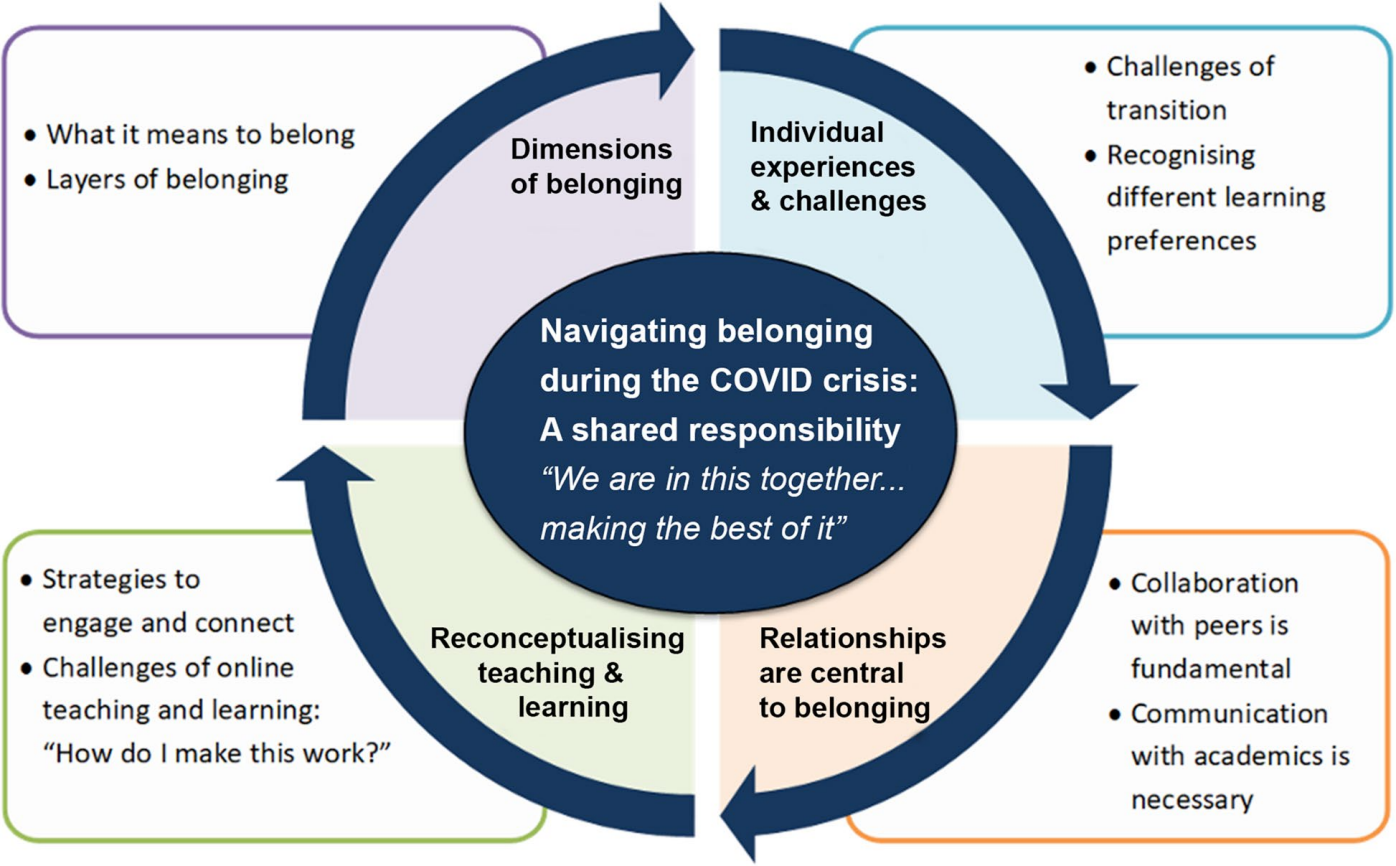
Pictorial representation of the global, organising, and basic themes

The shared experiences about *navigating belonging during the COVID-19 crisis*, have been captured under four organising themes: *dimensions of belonging*, *individual experiences and challenges*, *reconceptualising teaching and learning*, and *relationships are central to belonging*. Within each organising theme, basic themes were identified that provide depth to the organising theme (Fig. 1). Additional files 1 and 2 present a summary of the quotes obtained from the open-ended surveys and focus groups respectively, that contribute to the themes in Fig. 1.

#### Organising theme: dimensions of belonging

This theme outlines that belonging is a multidimensional experience with several facets underpinning participants’ experiences. Students and academics identified several dimensions of belonging in relation to first year students’ experiences, as illustrated by two basic themes that sit under the organising theme: *what it means to belong*, and *layers of belonging*.

#### *Basic theme: what it means to belong*

This theme explores the idea that belonging at university is underpinned by feeling valued and connected. Academics and students agreed that having a sense of being valued by the university and a desire to have an active connection across all aspects of university life was important for students.

Belonging as a student was gained through a connection with the “vocation” (Student-Claire-Focus Group) or the course and career, and with people who will “be there” (Student-Claire-Focus Group) for them. Furthermore, support of academics was critical to gaining a sense of belonging. It was noted by academics and students, that when students feel they belong at university, they are actively engaged in their learning, and this sense of belonging in turn shapes their overall identity. Students can then “actually sort of relax and become themselves” (Staff-Brooke).

Belonging to their cohort, their course, their future profession, and their university was important for students. One academic noted that the “concept of acceptance” is part of the sense of belonging and goes “both ways” (Staff-Brooke).

Both academics and students agreed that the rapid change to online learning due to COVID-19, meant that developing a sense of belonging was challenged.

#### *Basic theme: layers of belonging*

This theme identified layers of belonging reflected in participants’ experiences. Peer, academic and professional layers each contributed to an overall sense of belonging and key examples are provided below.

#### Peers

Belonging to peers was described as *“having that connection to someone that’s going through exactly the same thing as what you’re going through”* (Student-Astrid-Focus Group). Students were concerned that when learning moved online that this sense of belonging would be jeopardised by less opportunities for in-person interaction.

#### Academics

Being connected to academics was perceived by students as directly impacting learning, with one student commenting: *“…when they’re not connecting with the teacher, they’re not connecting with the content, they’re not connecting with the feedback. That’s when you develop this sense of feeling like you just don’t belong”* (Student-Emily-Focus Group).

Academics perceived it was also important for students to develop a sense of belonging to the university community.

#### Profession

Belonging to a profession was identified as an important feature of belonging by academics and students. Studying a degree with a clear professional identity facilitated first year students to feel they belonged compared to those undertaking general health science degrees which may have multiple pathways and career options less directly aligned to first year studies.

One academic actively encouraged first year students to belong to their professional association as a way of fostering belonging in first years.

#### *Organising theme—*Individual* experiences and challenges*

This theme outlines that while there are similarities in participants’ experiences, individuals have unique contexts and factors shaping their experiences. Academics and students reflected upon personal impacts of the COVID-19 pandemic on their teaching or learning and how they responded as individuals to the ensuing challenges. Two basic themes emerged: *Challenges of transition* and *recognising different learning preferences.*

#### *Basic theme—challenges of transition*

This theme explored the significant challenges of transitioning to online teaching and learning. For some students, the transition to online learning offered potential benefits of flexibility and reduced travel time. Two of the four students in the focus groups opted for online learning opportunities available in other subjects of study prior to the pandemic to efficiently manage their study and external commitments. Nonetheless, the pandemic brought a raft of personal challenges that diminished these expected benefits. Covid-related changes to family employment, reduced access to childcare support and non-optional home schooling presented new concerns.

Clearly, students missed the opportunity to focus attention on their learning needs when balancing childcare demands and home-schooling during lockdowns.

Unlike a conventional online courses where students choose or plan to be online, the sudden, unexpected, and unplanned move to online study was prefaced by a short period (four weeks) of in-person class time. This initial in-person time was identified as being key to relationship building.

Academics identified positive experiences and challenges during the transition to online learning. The rapid change presented a problem to be solved and individuals could “embrace it and to work effectively…as a team” (Staff-Jane). Quickly strategizing and responding to the demands of online learning required team knowledge, experience, and support. Hence, enhanced team culture was a further positive for academics, being “present for each other” (Staff-Brooke).

#### *Basic theme: recognising different learning preferences*

This theme identifies experiences of online learning influenced by personal attributes, individual expectations and learning preferences. Such key factors impacted students’ capacity to maintain focus on academic goals after the rapid change to online learning. Some students reflected that barriers were not solely a feature of online learning environments, reporting that competing priorities, including work commitments and limited contact time with staff as pre-existing challenges to belonging. However, some students directly attributed their limited engagement and reduced motivation to the online learning environment.

Students suggested that active engagement “comes down to personality” (Student-Astrid- Focus Group). If a student was not shy they were comfortable to come forward and participate online. Some students perceived clear links between personal discipline, engagement, commitment, and achievement in online learning environments.

Further, students perceived effective (and ineffective) online group functioning reflected personalities of individual members, with some groups/personalities seen as being able to organise whilst other groups lacked leadership and cohesion.

Students who perceived themselves as active engagers reported being drawn towards other students who demonstrated motivation to interact and learn. Other students perceived their personalities or learning preferences were misaligned with the expectations of belonging in online learning environments and focussed upon tasks rather than connection.

Academics recognised student diversity and a need to reflect and re-evaluate expectations of students in online environments. They accepted that some students may be quietly engaging and learning to belong, but this was harder to observe in online compared to in-person learning environments.

#### Organising theme—relationships are central to belonging

This theme identified the relationship between all parties as a fundamental aspect of creating a sense of belonging. Two basic themes were influential in shaping perceptions of how relationships and connections contribute to belonging: *collaboration with peers is fundamental*, and *effective and regular communication with staff is necessary*.

#### *Basic theme—collaboration with peers is fundamental*

This theme revealed collaboration with student peers was a key element of creating a sense of belonging. The degree of social interaction with student peers and opportunities to create friendships contributed to feelings of belonging. Accordingly, students found it problematic when peers neglected to turn cameras on during classes, making interaction very difficult. Visualisation of peers and use of cameras in online classes impacted students’ opportunities to get to know each other.

Challenges posed by online learning were further highlighted in the student survey through a focus on non-academic aspects of university and campus life. Typically, university campuses offer interactional opportunities through clubs, sport, and shared spaces to learn and socialise. Campus life, students suggested, may facilitate learning and personal development. Absence of this type of interaction was linked to barriers in developing friendships and consequently a lesser sense of belonging as reflected in Additional file 1.

#### *Basic theme—communication with academics is necessary*

This theme outlined that communicating with academics was a key component of creating a sense of belonging. With less opportunities for peer support, there was stronger reliance on the academic-student connection, although students reported positive and negative interactions with academics during online learning.

Positive interactions and individualised communication with academics enhanced student sense of satisfaction and belonging. Furthermore, students in the focus groups reported a feeling of trust and a bond created by a shared challenge. Survey responses echoed this sentiment, noting that academics were “non-judgmental and supportive” (Student Survey 18) and created a sense of camaraderie. However, when students perceived impersonal communication from academics, they felt less connected or believed that teaching had become a “transaction” (Student-Astrid- Focus Group). Perceived levels of enthusiasm and engagement from academics influenced student’s perceptions of connection and belonging.

Students identified the online environment as a barrier to communication with academics. While systematic and university level communication was perceived as a useful source of information, students prioritised individualised communication from academic staff as key to belonging.

Academics concurred that effective communication was challenged in online environments, missing non-verbal cues and responsivity that characterises a classroom environment. Although the online learning environment provides an opportunity for academics to connect professionally with students, there were students who left their cameras off, with one academic noting they didn’t push this issue because there are many reasons for students choosing this option.

#### Organising theme: reconceptualising teaching and learning

This theme reveals how academics and students reconceptualised their expectations and modes of teaching and learning, to manage the crisis. It was not easy for academics or students, and many strategies were employed to make it work, with two basic themes emerging: *challenges to online teaching and learning,* and* strategies to engage and connect.*

#### *Basic theme: challenges of online teaching and learning: “how do I make this work?”*

This theme outlined many challenges faced by both academics and students during a rapid change to online mode. With the rapid change to online learning, academics asked themselves, ‘How do I make this work?’.

#### Managing workload

Academics reported their workload increased significantly, and they “found it a juggling act” (Staff-Louise) to meet their teaching requirements. Administrative loads consequently increased when reduced in-person contact with students led to more electronic communication. Academics needed to up-skill in online teaching in a short time frame and perceived this responsibility as all encompassing.

The rapid switch to online learning attracted significant academic workload, implementing and adapting content to see how material “might play out in a Zoom environment…[where]…everything takes longer” (Staff-Natalie).

Some students noticed a temptation to disengage from online learning, which meant balancing their workload and study demands became a challenge as they also faced significant workload and stressors in their personal lives due to COVID-19.

#### *Class *dynamics

Academics and students spoke about the change to classroom dynamics. The online environment was noted as being one in which it was difficult to read the room to see how students were progressing with their work. Others tried to use humour to enliven a class, only to have the Zoom frame freeze, killing the mood they were trying to create. Hence, staff felt teaching online was less conversational, flexible and responsive compared to face-to-face. Moreover, academics missed hands-on practical elements; a big shift for some programs.

#### *Technological *challenges

Academics learnt new skills quickly, but often these skills would be challenged when technology failed. Some academics reported a sense of vulnerability due to technological ineptitude but acknowledged that making mistakes in front of students could humanise the experience. Academics also acknowledged that some students did not have adequate technological resources to meet changes in their learning requirements when classes were placed online.

#### *Basic theme: strategies to engage and connect*

This theme reflected the strategies academics and students employed to remain engaged and connected. Academics worked hard to enhance online learning and hoped to connect with students and engage them in activities. Students too were active and appreciated academics' efforts to facilitate engagement and connection. Underlying many of the strategies adopted by academics was a deep concern for student welfare during this time. Therefore, many academics aimed to ensure students were engaged and connected with each other and with the academic team. Academics built in small group opportunities during online teaching so students could connect, learn, and socialise.

Staff also spoke about informing students they could contact staff for support. One staff member described crossing the divide and actively discouraging a ‘them and us’ dynamic between students and staff.

A variety of teaching tools were identified by staff to build connection and promote engagement. Such tools included interactive quizzes, ice breakers activities, integrating reflective practices into activities and ‘drop in’ sessions. Staff also encouraged students to establish social media groups or other group experiences outside the classroom. Some staff members arrived early to zoom classes and left late to enable students to connect informally.

Students appreciated staff attempts to provide these activities. Students found these initiatives helpful, recognising staff placed effort into knowing students personally and focussing on student wellbeing and achievement. Students cited examples of provision of extra resources, mini-lectures, additional question and answer sessions, and fast response times to student queries. Students also initiated their own engagement strategies, including using group and personal messaging over platforms such as Facebook messenger.

## Discussion

Sense of belonging is a social construct predicated on cultural and social interactions of learners, peers, and academics; similarly, in a move from in-person to online learning, it is the social aspects that are often detrimentally impacted. Due to these foci, we used SLT [21, 27] to conceptualise student and academic perspectives on sense of belonging gained through our mixed methods study following a rapid move to online teaching. As discussed earlier, SLT focusses on social aspects of the learning process, through legitimate peripheral participation (LPP) rather than a cognitive motivation and importantly has previously been a useful framework in similar settings considering both students [22, 24] and academics [36]. Our key finding reiterated through comments from the student survey and both student and academic focus groups was consolidated into our global theme “We are in this together…make the best of it”. As this epitomises the essence of sense of belonging, to be accepted and part of a community [37], we could interpret this as demonstration of a strong sense of belonging among participants, but it is the latter part of the theme, “make the best of it” and the layers of belonging identified in the organising themes explored in Additional files 1 and 2 that demonstrate the complexity.

Using the SLT framework enabled us to consider the importance of the social aspect of learning and the role relationships play in learning, but also went beyond this to consider the unintentional learning that may have been lost in the rapid transition online [21]. Our findings that students without previous higher education experience were more prone to consider leaving the course underscored the importance that relationships and connections had in belonging. Although not a direct correlation, similar results have been seen previously in relation to maturity playing a role in positively influencing an individual’s sense of belonging and retention [38].

We are aware that some themes identified may be considered artefacts of the COVID-19 pandemic and the associated rapidity of the move to online teaching and learning. Nonetheless, our findings align with previous commentary in this area and contribute valuable insights for the clinical health sciences student population, reinforcing the continued need for study into how to address and develop an online sense of belonging [6, 9, 39].

Belonging is clearly more than a unidimensional concept that can be given to or received by students. Comments from both academics and students corresponded to previous definitions of belonging [3] indicating a connection to something bigger or a sense of acceptance. In our study, the connection refers to the layers of belonging including peers, academics, university, and the profession. The relationships between these layers were central to belonging. Our students noted that connections needed to be genuine rather than tokenistic, preferring to be known as an individual even if it was at a more local level, in accordance with the findings of Yuan & Kim [40]. Understandably then, given the barriers to physical interactions, we found it was this student connectedness that was most drastically impacted compared to the connections to university, prospective profession, and staff. This peer connection identified as fundamental by students in developing a sense of belonging and conceivably developed through LPP within an on-campus learning environment [22], will become the crux of fostering a comprehensive sense of belonging in an online environment. Concerningly, our findings suggested that when students log on for a class, the opportunity to engage in any form of unintentional learning experience was immediately replaced with a purposeful interaction bracketed within a finite timeframe and negating opportunities for socialisation or LPP.

Our findings indicated students gained a strong understanding and knowledge of their future professions suggesting that online classes met some of the needs of knowledge transfer and development, but it was the serendipitous connections before, after and between classes that no longer existed and, their absence limited a pivotal social aspect of belonging [9]. Consequently, the academic-student relationship became more important, developing into a proxy peer relationship for some students who had no other peer supports. The key factor, from both academics and students, was the need for reciprocity in the development of a sense of belonging, a finding consistent with Mahar and colleagues in their 2013 review to conceptualize belonging [3]. An unexpected finding was the way in which the rapid move to the online both facilitated and hindered reciprocal engagement. Clearly, academics and students perceived the rapid transition to online learning threatened the co-construction of learning culture during the first stages of health science education. This threat gave both academics and students a distinct sense of “being in it together” which led to a breakdown of many typical student–teacher barriers; an experience which has been evidenced worldwide [41]. In this regard, sense of belonging was being developed as roles of support and co-construction of knowledge, a central tenet of SLT [22], moved between student and academic and the concept of the classroom became more amorphous.

Logistical and infrastructural complexities to place of learning and belonging were identified through the organising theme: individual experiences and challenges. With the loss of the physical learning space, came incremental impacts from other aspects of life (family and work) and consequently erosion of the opportunity to develop a sense of identity within a community and through that a sense of belonging. Importantly, this incursion into non-university life was highlighted by both academics [42] and students. In students, reduced access to a shared physical learning space, led to a decrease in sense of belonging and in academics it resulted in increased workload and a sense of exhaustion. Such negative effects could conceivably lead to academic disengagement and further impact student sense of belonging during prolonged learning experiences [43]. Indeed, life outside university and individual personality factors impacted developing connections and sense of belonging irrespective of where learning took place. SLT espouses the concept that learning is a social experience, one built on relationships, where those more experienced support those still developing [27]. Yet clearly socialisation requires extra effort or overt participation as one student noted. Many students supported the views of previous studies [44, 45] in noting that they did not have the time nor energy to participate in learning to this level and therefore, although they acquired content knowledge, the opportunity for developing a sense of belonging was lost. This lack of engagement was by no means universal amongst students. However, without the incidental connections that are made through LPP outside the classroom by those who were engaging, students struggling to engage may be further disadvantaged.

An argument may be made that the external life influences during the COVID-19 pandemic are not representative of the normal university experience and therefore impact transferability of our themes. The pandemic presented innumerable challenges. However, the transition from school to university, or university to the workforce, is always a tumultuous time representing many firsts [46, 47]. As such, we argue that the challenges and responses identified through our research will remain relevant beyond the context of a pandemic and present an important opportunity for future educators and researchers to address. Nonetheless, significant challenges provided opportunities for creating a learning culture grounded in motivation to adapt and achievement of learning outcomes.

Despite the significant challenges occurring with the rapid transition to online learning, academics and students rose to the challenge and reconceptualised learning and teaching processes. Indeed, this reconceptualisation of the learning and teaching process and strategies to connect were evident as organising and basic themes, including reconceptualising teaching and learning and a focus on how to make the teaching work. One of the greatest hurdles presented was a decrease in spontaneity of the experience, either due to a lack of visual cues or technological lags [48]. These hurdles were addressed through both pedagogical and technological means, changing class sizes, having consistent student groups, and using myriad technology and apps. Although students and academics acknowledged this work, the level of connectedness was perceived as stilted and the ability for reciprocity was limited [13]. These changes indicate the willingness of academics to trial, evaluate and adapt their teaching (and technology) to achieve not just knowledge transfer, but also a sense of belonging. Identifying hurdles that were more difficult to surmount may also facilitate academics in the development of strategies to better manage future rapid transitions to online learning, an ongoing potential scenario in higher education. Optimistically, the findings from this study suggest that future innovations, and academics’ creativity and willingness to trial interventions, may overcome some of the hurdles to sense of belonging that we have identified.

## Limitations

As with any study, our research had some limitations, and the results should be considered in light of these. We had ~ 27% of the cohort complete the student survey, possibly due to survey fatigue associated with multiple other requests for data during this tumultuous period. In saying so, the demographic characteristics of students who responded were reflective of the size and sex makeup of the first year cohort of each clinical health program. The academic focus group included five participants ranging from full time to casual academics, but the student focus group included only four students and was skewed toward more mature students rather than school leavers. While this could have impacted the qualitative results, the themes from the student focus groups were supported by the qualitative responses gained through the open-ended questionnaire data which included responses from a larger more diverse sample of students.

## Conclusion

While the rapid transition to online learning did not greatly impact first-year students feeling respected and welcomed at the university, the lack of connectedness with peers and academics during this experience has affected their overall sense of belonging. The ability to initiate and maintain connectedness with peers and academics was considered integral and sparked creativity among academics and students to reconceptualise their learning and teaching approaches. However, maintaining an overall sense of connectedness has been, and will continue to be, challenging due to the complexities faced at each level of belonging. Through clarifying perceptions of belonging, and hurdles faced by both academics and students in its development, we can create better strategies targeted at fostering a stronger sense of belonging among first year clinical health science students.

## Supplementary Information

**Additional file 1.** Selected illustrative quotes from the open-ended survey responses arranges in themes.

**Additional file 2.** Selected illustrative quotes from student and staff focus groups.

## Data Availability

The data that support the findings of this study are available from Western Sydney University, but restrictions apply to the availability of these data, which were used under license for the current study, and so are not publicly available. Data are however available from the authors upon reasonable request and with permission of Western Sydney University Human Ethics Committee.

## Abbreviations

CI: Confidence interval
COP: Community of practice
*df*: Degrees of freedom
LPP: Legitimate Peripheral Participation
IQR: Interquartile Range
OR: Odds ratio
SD: Standard deviation
SLT: Situated Learning Theory
