# Supplementary material for: Impact of online learning on sense of belonging among first year clinical health students during COVID-19: student and academic perspectives
Source: BMC Med Educ. 2023 Feb 8;23:100. doi: 10.1186/s12909-023-04061-2 (PMC9906584; doi:10.1186/s12909-023-04061-2)
Supplement: Supplementary file 1 — Additional file 1. Selected illustrative quotes from the open-ended survey responses arranges in themes. [file 12909_2023_4061_MOESM1_ESM.docx]

**Additional File 1:** Selected illustrative quotes from the open-ended survey responses arrange in themes (n=127 surveys)

| **Global theme**  Navigating belonging during the COVID-19 crisis: A shared responsibility “We are in this together...making the best of this” | |
| --- | --- |
| **Organising theme:** Dimensions of Belonging | |
| **Basic themes** | |
| Layers of belonging:  peers, academics, university, profession | Well, I haven't really felt like I belong as I lack the connections and associations with other students and academic staff to feel like I am a part of my online course. The greatest sense of belonging I have felt is the getting an exam back that I did well in but really it wasn't much*. (Student survey 175 – 18-24 years)*    I met two people doing the same course as me and got their social media info. So, when the transition to online came about, I already had friends I could talk to. That is the only thing that has made me feel I belong*. (Student survey 72 – 18-24 years)*    Transitioning to online classes have largely affected my sense of belonging in my first semester. I feel disconnected from my course as well as my peers*. (Student survey 91 – 18-24 years)*  The connection between all students, knowing we are all in this course for one main reason; to be the ones to have a positive impact on an individual’s life and help them with our greatest potential *(Student survey 34 – 18-24 years)* |
| **Organising theme:** Individual experiences and challenges | |
| **Basic themes** |  |
| Challenges of transition | Feeling disconnected from the content and the university. *(Student survey 180 – undisclosed age)*    This is a tricky one, to be honest I don't think I really did feel a sense of belonging and felt quite out of my depth. While I definitely support the transition to online learning, I think initially there was a big disconnect and it was much harder to become and stay engaged with both other students and academic staff. *(Student survey 156 – 18-24 years)*    Now with online classes, I do not know anyone or speak to anyone and hardly attend classes because I cannot focus or ask questions. One of my tutors made me feel guilty for not attending online tutorials. But I really hate online learning. I just love face to face classes, I feel more engaged and can ask questions freely. I felt that I wanted to avoid more classes because of the way my tutor dealt with my unattendance. *(Student survey 9 – 18-24 years)* |
| Recognising different learning preferences | My age [mature age student] and the distance that I live from university I believe have a greater impact on my ability to form relationships with other students than the online learning aspect of the courses at the moment. *(Student survey 10 – 30-34 years)*    Online - No motivation…Not engaging. Even the teachers sound asleep. I could listen to a 10hr lecture and still feel like I’ve learned nothing. *(Student survey 100 – 18-24 years)* |
| **Organising theme:** Relationships are central to belonging | |
| **Basic themes** |  |
| Collaboration with peers is fundamental | Majority of people don't even have their cameras on in online classes so even the people you do connect with you won't recognise in person lol. Hence it is hard to connect therefore feel a sense of belonging. *(Student survey 65 – 18-24 years)*    …making any friends. Not being able to see faces of the other students during class time. Not having any social time to interact. *(Student survey 8 – 18-24 years)*    Lack of friendships have made it difficult, as we no longer have anyone to talk to about assessments/exams to see if we are on the right track. *(Student survey 57 – 30-34 years)* |
| Communication with academics is necessary | There have been a few personal obstacles to study that I would have liked to discuss with the teachers but am not comfortable formalising into an email or discussing in an online class. *(Student survey 13 – 18-24 years)*    I like how staff seem to know students personally and strive to seek our wellbeing and best achievement. *(Student survey 83 – 18-24 years)*    Classes are more accessible and flexible online. It’s much easier to communicate with staff. Everyone is on the same level and the groups change all the time moving to break out rooms allowing for me to meet more people. *(Student survey 161 – 18-24 years)* |
| Challenges of online teaching and learning: “How do I make this work?” | This online study especially to me is total mess. Having baby at home it is impossible to concentrate as there are too many distractions. And limited time for each classes have made it harder to interact, discuss with teacher and communicate with other friends*. (Student survey 139 – 18-24 years)*    Work commitments, limited contact time with staff as well, and also lack of response by… students if you post something in the Facebook group page. *(Student survey 45 – 18-24 years)*    Everything is online, there are no team assessments, everything you do is solitary. Most people don't have cameras on in tutorials so it feels like there are no other students. Due to COVID, study was done at home, so there was no group study at a library or anything like that. *(Student survey 112 – 25-29 years*) |
| Strategies to engage and connect | The unit facilitator really excellent in making sure he was accessible through the Discussion board on vUWS and the weekly collaborate sessions. Through having this access to support definitely impacted my sense of belonging as student for this unit*. (Student survey 130 –undisclosed age)*    Working in break out rooms in my course specific subject and interacting with those with who want to work with each other and communicate. *(Student survey 118 – 40-44 years)*    I met two people doing the same course as me and got their social media info. So when the transition to online came about, I already had friends I could talk to. That is the only thing that has made me feel I belong. *(Student survey 72 – 18-24 years)*    Non-judgemental and supportive role played by some of my unit coordinators and tutors, particularly within population health and society. This helped to lessen the stress of feeling that I am not keeping up or that I am underperforming. Access to disability support staff, and services provided to support differently abled students helped support my belonging. In particular, I attended a webinar about IT tools and study strategies and it was nice to know everyone struggles with different aspects of study. Staff members described their own challenges which also helped to me feel like less of a failure and helped to normalise my experience. I enjoyed that I could watch and listen without identifying myself and feeling singled out. *(Student survey 18 – 18-24 years)*    The respect from the academic staff in my course. students have made a Facebook group and we are connected through that *(Student survey 1 – 18-24 years)* |
