# Supplementary material for: Impact of online learning on sense of belonging among first year clinical health students during COVID-19: student and academic perspectives
Source: BMC Med Educ. 2023 Feb 8;23:100. doi: 10.1186/s12909-023-04061-2 (PMC9906584; doi:10.1186/s12909-023-04061-2)
Supplement: Supplementary file 2 — Additional file 2. Selected illustrative quotes from student and staff focus groups. [file 12909_2023_4061_MOESM2_ESM.docx]

**Additional File 2:** Selected illustrative quotes from student (n=4) and staff (n=5) focus groups

Pseudonyms used throughout

| **Global theme**  Navigating belonging during the COVID-19 crisis: A shared responsibility “We are in this together...making the best of this” | |
| --- | --- |
| **Organising theme:** Dimensions of Belonging | |
| **Basic themes** |  |
| Student perspective  What it means to belong? | At the end of the day, um, for me uni is a vocation, and – and the people who are in the uni is what brings me in there; that they’re the people that…want me to go to uni…I know I can go there and there will be someone there that I can talk to. *(Student – Claire)*    I think that was the thing that struck me straight away, was how welcoming and supportive I felt they were.  Like, I don’t know what I was expecting uni to be like, but I feel like the tutors really care and just want you to feel really supported. *(Student – Astrid)* |
| Staff perspective  What it means to belong? | I guess…probably just feeling like you’re a part of something.  Maybe a part of something bigger…having commonalities with…other people that you’re studying with...I guess, belonging to…the course…belonging to university. *(Staff – David)*    …acceptance in the sense of that perhaps as an individual, the student feels that they are…valued and acknowledged within the community, whether it be their…professional community, their class community or the university community as a whole.  But also, that they also see something within those communities that actually attracts them, or really aligns with their values or their aspirations, so that they have that motivation to want to be part of that group as well. *(Staff – Brooke)* |
| Student perspective Layers of belonging: peers, academics, university, profession | ...they [academics] stressed that we rely on peers to support each other because we do go through some challenging things...so they were emphasising how important it is that we have each other and have each other’s backs…And they were like…“You’ll get to know everybody”, and then COVID sort of hit, and...I remember just being disappointed.  I was like, how am I meant to...meet everyone and create that?” *(Student – Claire)*    ...when they’re not connecting with the teacher, they’re not connecting with the content, they’re not connecting with the feedback. That’s when you develop this sense of feeling like you just don’t belong *(Student – Emily)*    ...clinical specific students…can identify…who they want to be when they graduate. So that identity, I think, would really make a difference in that sense of belonging, especially with the online [learning].*(Student – Emily)* |
| Staff perspective Layers of belonging: peers, academics, university, profession | ...there’s several layers of it...and it sort of radiates outwards, maybe from the immediate core sense of belonging or professional sense of belonging into those wider...university areas and more and more people as well...the immediate peers, but then educators and then, other sort of departments within the university as well. *(Staff – Lydia)*    I think that’s important to feel part of the bigger university picture and university experience as well *(Staff – Natalie)* |
| **Organising theme:** Individual experiences and challenges | |
| **Basic themes** | |
| Student perspectives  Challenges of transition | It was the transition outside as well…worrying about, you know, am I going to have enough money to feed my family…Am I going to have internet that works to be able to attend my classes...My headphones are on, mute and I’m screaming, “Get off the internet.  Mummy needs the internet” *(Student – Emily)* |
| Staff perspectives  Challenges of transition | …embrace it and to work effectively…as a team *(Staff – Jane)* |
| Student perspectives  Recognising different learning preferences | We’re a different personality and we turn up and we do all the work and stuff, like, we set ourselves up for success.  I just really feel for the people that are struggling with that and…don’t have the same kind of discipline. *(Student – Sarah)*    Where some of the groups fell down is where they didn’t have the mature-age student in the mix, or they didn’t have a dominant personality that was able to cohesively organise them. *(Student – Emily)*    Developing a sense of belonging in the online environment seemed to require overt participation in online learning activities. This was contrary to my 'keep your head down' approach to learning. For this reason, I personally…did not prioritise acquiring a sense of belonging*. (Student – Emily)* |
| Staff perspectives  Recognising different learning preferences | …it’s recognising different learning preferences and…some students’ preferences to actually be a little bit quieter and a little bit in the wings…and I’ve heard…that they are highly, highly engaged students. *(Staff – Lydia)* |
| **Organising theme:** Relationships are central to belonging | |
| **Basic themes** | |
| Student perspectives Collaboration with peers is fundamental | I had a really great first semester…I was in one particular tutorial where I had really like-minded people. *(Student – Sarah)*    The people that I go to uni with…definitely helped me with the sense of belonging. *(Student – Claire)* |
| Staff perspectives Collaboration with peers is fundamental | Every week, they had the same 10 people in their breakout room and they got to make some friends...so at the end of second semester, they’ve actually come out with knowing five really close friends, which I think is an achievement *(Staff – Natalie)* |
| Student perspectives Communication with academics is necessary | Our tutors did a really good job at making sure like we belonged, um, like, in those first few weeks that we were on campus but even more so, probably, while we were in Zoom…I thought we’d go into class and you’d not really feel like you have a relationship with the tutor, but they’re just so caring and lovely, and you feel – well, I feel like I can definitely go to them for anything if I had an issue…and they would help. *(Student – Astrid)*    Getting emails from [the University] saying, like, ‘We hope you’re all okay.’ Like, that is nice, but it’s more about people and having support from people or relationships with people.  Yeah. *(Student – Astrid)* |
| Staff perspectives  Communication with academics is necessary | We [academics] felt that that reciprocal exchange of communication as part of teaching activities really early on, would start to set good foundations for sort, um, effective teaching relationships that are with the students *(Staff – Lydia)* |
| Student perspectives Challenges of online teaching and learning: “How do I make this work?” | Am I going to have to put a screen up because my daughter wants to run past naked? *(Student – Emily)* |
| Staff perspectives Challenges of online teaching and learning: “How do I make this work?” | So really, I felt that teaching has been 24/7 this whole year and that has been quite exhausting…Learning has been hard too. I’ve had to learn Zoom. I’ve had to learn all different ways of…filming myself, filming classes, putting up…transcription, all of that kind of things. *(Staff – Natalie)*    Trying to get some form of feedback, did you understand my instruction?  Do you need some help?  Blank faces or black squares made it very hard for me to judge what I needed to change in a particular class to adapt to the students in that room at that time*. (Staff – Natalie)*  …it just didn’t have the same effect and so, having to just rely on words and text and slides, I felt like there was a big section of my teaching missing *(Staff – Louise)* |
| Staff perspectives Strategies to engage and connect | …a student…said to me, “Thank you for all your work this semester…I love the classes, but as soon as I leave the Zoom room, my world goes black”.  And that was really, really sad. *(Staff – Louise)*    across the tutorial, they get some opportunity to work individually on a task, some opportunity to work in pairs..., some opportunity to work in a little group of four…And I think, eventually students got used to that formula a little bit…I think that gives some familiarity and comfort and predictability as well. *(Staff – Lydia)*  So you’re there and if somebody comes early, they might want to have a chat…like, reciprocating what you would do…on campus, where you’re there beforehand. If you’re not going to have to rush off to another lecture, you’re kind of there outside the door as well…when you’re leaving…so being available on that platform, just seeing if anyone’s got any questions at the end. *(Staff – Jane)* |
